# Go4it; study design of a randomised controlled trial and economic evaluation of a multidisciplinary group intervention for obese adolescents for prevention of diabetes mellitus type 2

**DOI:** 10.1186/1471-2458-8-410

**Published:** 2008-12-16

**Authors:** Geesje H Hofsteenge, Marijke JM Chinapaw, Peter JM Weijs, Maurits W van Tulder, Henriette A Delemarre-van de Waal

**Affiliations:** 1 Dept. of Nutrition & Dietetics, VU University Medical Center, Amsterdam, The Netherlands; 2 EMGO Institute, VU University Medical Center, Amsterdam, The Netherlands; 3 Dept. of Public and Occupational Health, VU University Medical Center, Amsterdam, The Netherlands; 4 Dept. of Health Sciences, Faculty of Earth and Life Sciences, VU University, Amsterdam, The Netherlands; 5 Dept. of Paediatrics, VU University Medical Center, Amsterdam, The Netherlands

## Abstract

**Background:**

In the Netherlands, the first adolescents with diabetes mellitus type 2 as a result of obesity have recently been diagnosed. Therefore, it is very important that programs aiming at the prevention of type 2 diabetes of obese adolescents are developed and evaluated.

**Methods:**

Go4it is a multidisciplinary group treatment that focuses on: 1) increasing awareness of the current dietary and physical activity behaviour (i.e. energy balance behaviour), 2) improving diet, 3) decreasing sedentary behaviour, 4) increasing levels of physical activity, and 5) coping with difficult situations. Go4it consists of 7 sessions with an interval of 2–3 weeks.

The effectiveness of the multidisciplinary group treatment compared with usual care (i.e. referral to a dietician) was evaluated in a randomised controlled trial. We examined effects on BMI(sds), body composition, energy expenditure, glucose tolerance and insulin resistance (primary outcome measure), as well as dietary and physical activity behaviour and quality of life. An economic evaluation from a societal perspective was conducted alongside the randomised trial to evaluate the cost-effectiveness of the multidisciplinary treatment program vs. usual care.

**Discussion:**

In this paper we described a multidisciplinary treatment program (Go4it) for obese adolescents and the design of a randomised controlled trial and economic evaluation to evaluate its effectiveness and cost-effectiveness.

**Trial registration:**

Netherlands Trial Register (ISRCTN27626398).

## Background

Overweight and obesity in childhood are associated with negative psychosocial factors, orthopaedic complications, an increased risk for diseases such as hypertension, dyslipidaemia, asthma, sleep-apnoea and a strong increase of impaired glucose tolerance [1,2]. Impaired glucose tolerance precedes the onset of type 2 diabetes mellitus [3]. Overweight children are likely to remain overweight and are at increased risk of becoming obese compared to children of normal weight [4].

Sinha and co-workers found impaired glucose tolerance in 21% of obese children and 25% of obese adolescents [3]. Four percent of these obese adolescents had type 2 diabetes. At the paediatric outpatient clinic of the VU University Medical Center, glucose-intolerance and insulin-resistance was diagnosed in 18% and 52% of obese adolescents, respectively [5]. In 2003, the last Dutch national surveillance system of diabetes in children reported the first children with type 2 diabetes as a result of obesity.

Several reviews on interventions for treating obesity in children have been produced recently. These reviews suggest that there is some evidence that treatment of obesity in children and adolescents is feasible. However, there is a limited amount of data on effective components of programs to treat childhood obesity [6-10]. Cognitive behavioural modification techniques have shown promising results regarding lifestyle changes in obese children [11]. For obese adolescents the impact of an intensive intervention programme aimed at attaining a healthy lifestyle has not yet been evaluated. The prevalence of obesity in the Netherlands is, analogous to the United States, expected to increase in the coming years [12]. Therefore, there is an urgent need for interventions aimed at the prevention of obesity among adolescents as well as effective therapeutic interventions aimed at a healthy weight of the already obese.

For this reason, we developed Go4it, an intervention program for obese adolescents. This paper describes the design of the Go4it study, a randomised controlled trial (RCT) with an economic evaluation alongside. The aim of this study is to investigate the effectiveness and cost-effectiveness of this multidisciplinary group treatment for obese adolescents.

## Methods

### Design

The effectiveness of Go4it will be evaluated in a randomised controlled trial with an economic evaluation alongside. The Medical Ethics Committee of VU University Medical Center approved the study protocol.

### Intervention

Go4it is a multidisciplinary group treatment for obese children that is based on the programmes of Braet et al [11], Epstein et al [13] and the materials of the Dutch Obesity Intervention in Teenagers (DOiT) [11,14]. During 7 sessions with an interval of 2–3 weeks the adolescents received education on dietary behaviour, physical activity and energy balance. They also received cognitive behavioural therapy in which they were learned how to improve their lifestyle regarding a healthy weight and how to maintain an adequate energy balance. Go4it was carried out multidisciplinary in an outpatient clinic with a dietician, paediatrician/endocrinologist and psychologist involved. The first session was focussed on increasing awareness of the current lifestyle. Besides dietary and activity journals, step counters (pedometers) were used to increase awareness of the actual behaviour. Next, the adolescents had to set goals with respect to improving their energy balance behaviour. Additionally cognitive-behavioural therapy characteristics were used, e.g. learning to cope with difficult situations (parties, holidays) and teasing. Go4it works with homework tasks and the education is interactive. After 6, 14, 26, and 36 weeks, booster group sessions were scheduled in order to encourage the adolescents to maintain or further improve their energy balance behaviour and to discuss possible problems or questions. In addition, two separate sessions for the parents were organised. These parental sessions consisted of education concerning the health risks of overweight, healthy physical activity and dietary behaviour and how to support their children. Special materials were developed for this program; an information book, a workbook, and a dietary and activity diary. In addition, specific worksheets for every session were developed. The control group received the current regular care, consisting of referral to a dietician in the home care setting.

### Study population

#### Recruitment of the study population

All adolescents with overweight or obesity in the age range of 12–18 years who were referred to the outpatient paediatric obesity clinic of the VU University Medical Center were asked to participate. Overweight and obesity were defined according to the Cole criteria [15]. A paediatrician interviewed all adolescents concerning past medical history, weight development, anthropometrics, socio-economic status (SES) of the parents, and ethnicity. Additionally the following three questionnaires were administered as part of the regular care of the obesity clinic. The adolescents and their parents also received an information brochure about the study. Within two weeks, we phoned them to ask for their willingness to participate. Written informed consent was obtained from all adolescents as well as their parents.

#### Child Behaviour Checklist *(filled in by parents)*

The Child Behaviour Checklist (CBCL) is a questionnaire assigning quantitative scores to behaviour problems and competences of children and adolescents at 4 to 18 years of age. The CBCL was filled out by parents or other caregivers. It is composed of 20 competence items and 118 behaviour problem items grouped into four competence scales and eleven problem scales. The competence scales include: activities (amount and quality of participation in sports, hobbies, games as well as jobs and chores), social (number and quality of relationships with others and involvement in organizations), school (an index of competence in school) and total competence score obtained summing the activities, social and school scales (a global index of adaptive functioning). The problem scales include eight syndrome scales: withdrawal, somatic complaints, social problems, anxiety/depression, thought problems, attention problems, delinquent behaviour, and aggressive behaviour; two psychopathology scales, internalizing and externalizing and, lastly, a total problem score, an overall index of the reported problems [16,17].

#### Youth Self-Report *(filled in by the adolescents)*

The Youth Self Report (YSR) provides self-ratings for the same competence and problem items as those of the CBCL. The YSR also includes open-ended responses to items covering physical problems, concerns, and strengths. The adolescents rate themselves for how true each item is now or was within the past six months, using the same three-point response scale as the CBCL [16,18,19].

#### Child Eating Disorder Examination-questionnaire

The Child Eating Disorder Examination Questionnaire (ChEDE-Q)measures three forms of overeating: objective bulimic episodes, subjective bulimic episodes and objective overeating episodes. The ChEDE-Q also measures four methods of weight control: self-induced vomiting, laxative misuse, diuretic misuse and intense exercising [20]. The items of the ChEDE-Q are derived from the initial eating disorder examination interview [21]. The ChEDE-Q was modified to make it suitable for use in populations of Dutch children and is more concrete and simpler for children.

#### Inclusion and exclusion criteria

Adolescents are eligible when they meet the following inclusion criteria: 1) age between 12–18 years; 2) overweight or obesity according the definition of Cole et al [15]. Exclusion criteria for the study are: not Dutch-speaking, overweight/obesity as a result of a known syndrome or organic cause (hypothyroidism), mental retardation, physical limitations and diagnosed Diabetes Mellitus type 2.

### Randomisation procedure

All participants will be randomly assigned to the intervention or control group, using SPSS for a random selection of a sample. Randomisation was stratified by age group (12–14 y, 15–18 y) and gender. Randomisation took place after the baseline measurements.

### Sample size calculation

The sample size calculation is based on a difference in BMIsds of 0.29 (10%) between the intervention and control group after 18 months. The standard deviation for this BMIsds is considered to be 0.5. Based on these data, in an intention-to-treat analysis, 43 subjects per group are needed to detect this difference in mean BMIsds with an alpha of .05 and a power of .80. A sample of 108 persons (n = 54 per group) is required taking into account a dropout rate of 25% [5,11].

### Measurements

Primary outcome measures of the randomised trial was BMI(sds). Secondary outcome measures were glucose tolerance, dietary behaviour, physical activity, sedentary behaviour and self-esteem. Data were collected at baseline and after 6 and 18 months intervention. All measurements were performed after an overnight fast, except for the dual energy x-ray absorptiometry (DEXA).

### - Body composition

#### Height, weight and waist circumference

Height was measured and recorded with an accuracy of 1 mm with a electronic stadiometer (KERN 250D). Body weight was measured (in underwear) and recorded within 0.1 kg with a calibrated electronic flat scale (SECA 861). Weight and height were used to calculate BMI (weight in kilograms divided by the square of height in meters). The degree of overweight was quantified using Cole's least mean square method, which normalized the BMI's skewed distribution and expressed BMI as BMIsds [15]. Reference data of Dutch children were used (; versie 3.5). The BMIsds indicates how many standard deviations a measurement is above or below the median of the distribution.

#### Bio-electrical impedance spectroscopy

After an overnight fast, bio-electrical impedance spectroscopy (BIS) measurements were performed at the non-dominant side of the subject, using a Hydra ECF/ICF Bio-Impedance Spectrum Analyzer, model 4200 (Xitron Technologies, San Diego, CA, USA). Shoes and socks were removed and the subjects were in a supine position. Two current electrodes (tetra-polar electrodes (3 M red Dot AG/AgCl)) were placed at the dorsal surfaces of the hand and foot on the distal portion of the second metacarpal and metatarsal, respectively. Two detector electrodes were placed at the posterior wrist between the styloid processes of the radius and ulna and at the anterior ankle between the tibial and fibular malleoli. The resistance and reactance measured at 50 kHz were used in the analysis, obtained by the program Hydra Data Acquisition Utility. With this technique, two body compartments, fat mass (FM)) and fat free mass (FFM), can be determined.

#### Dual energy x-ray absorptiometry

Subjects were scanned for 10 minutes in underwear while lying in supine position with arms and legs abducted from the body. All measurements were whole body scans performed with a Hologic QDR-Delphi, S/N 45665. The DXA method measures three components of the body: bone mineral content (BMC), fat mass (FM) and lean tissue mass (LTM). These components were calculated based on the differential attenuations of the two photon beams, caused by the differences in density and chemical composition [22,23].

### Energy expenditure

Resting oxygen consumption (VO_2_), and carbon dioxide production (VCO_2_) were measured by O_2_, CO_2 _flow with a ventilated hood system for 30 minutes with a metabolic monitor (Vmax Encore n29, Viasys Healthcare, Houten, The Netherlands) after an overnight fast. Before each test, the gas analysers were calibrated with two reference gas mixtures (16% O_2_, 4% CO_2 _and 26% O_2 _(Viasys)). The subjects were monitored during the measurements to prevent any movement or sleeping under the hood. The first 5 minutes were excluded to account for environment adjustment by the children and gas adaptation in the hood. Then resting energy expenditure (REE) was calculated from oxygen consumption and carbon dioxide production by using the equation of Weir [24].

### Metabolic measurement

#### - Glucose tolerance and insulin resistance (Oral Glucose Tolerance Test)

After an overnight fast subjects were given glucose, in a dose of 1.75 g per kilogram of body weight (up to maximum of 75 g) orally, and blood samples are obtained after 0, 30 and 120 minutes for the measurement of plasma glucose and insulin levels. Impaired glucose tolerance was defined, according to the American Diabetes Association guidelines, as a fasting plasma glucose level between 5.6 – 6.9 mmol/l and a two-hour plasma glucose level between 7.8–11.1 mmol/l. Type 2 diabetes was defined as a fasting glucose level of 7.0 mmol/l or higher or a two-hour plasma glucose level of more than 11.1 mmol/l [25].

### Questionnaires

After the Oral Glucose Tolerance Test (OGTT) participants complete the following questionnaires:

#### - Dietary behaviour

Questions about two risk behaviours i.e. consumption of sugar-sweetened beverages and consumption of high-sugar/high-fat content snacks [14]. The questionnaire is based on other validated questionnaires for assessing dietary intake [26,27]. Adolescents indicate how many days a week they consume sugar-sweetened beverages or energy dense snacks, and the amount/number of servings of sugar-sweetened beverages or energy dense snacks they usually consume on these days, thinking of the last week.

#### - DEBQ: Dutch Eating Behaviour Questionnaire

Eating behaviour is assessed using the Dutch Eating Behaviour Questionnaire. This questionnaire consists of 33 items, on emotional (13 items), external, and restrained eating (both 10 items). All items are rated on a five-point scale ranging from 1 (never) to 5 (very often). The DEBQ scales have high internal consistency, high validity, and high convergent and discriminative validity [28,29].

#### - Physical activity and sedentary behaviour

The Activity Questionnaire for Adolescents & Adults (AQUAA), is based on the Short Questionnaire to Assess Health-enhancing physical activity (SQUASH)[30]. This questionnaire was not designed to asses' energy expenditure, but to give an indication of the habitual activity level and sedentary behaviour. The AQuAA questionnaire is structured in commuting activities, leisure time activities, household activities, and activities at work and school. For every item examples of activities are provided. The test-retest reliability and validity are currently being studied (Chin A Paw et al, submitted).

#### - Quality of life

##### Child Health Questionnaire (CHQ)

The 87-item questionnaire-child form is a generic health status measure covering physical and psychosocial domains and was developed specifically for children and adolescents. The 87 items with 4, 5 or 6 response options is divided over 10 multi-item scales and two single-item questions. Per scale the items are summed up and transformed into a 0 (worst possible score) to 100 (best possible score) scale [31,32].

##### Pediatric Quality of Life Inventory™ Version 4.0 (PedsQL™ 4.0)

The 23-item PedsQl ™ 4.0 Generic Core Scales for children aged 2 to 18 years encompasses:

1) physical function (8 items), 2) emotional functioning (5 items), 3) social functioning (5 items), and 4) school functioning (5 items). The instrument takes approximately 5 minutes to complete. The instructions ask how much of a problem each item has been during the pas month. A 5-point Likert response scale is utilized (0 = never a problem to 4 = almost always problem). Scales are standardized, and scores range from 0–100, with higher scores representing better quality of life. The PedsQL has been shown to be both reliable and valid [33,34].

##### Body Esteem Scale for Adolescents and Adults

The body esteem (BE) questionnaire for adolescents has 3 subscales: BE-Appearance (general feelings about appearance), BE-Weight (weight satisfaction), and BE-Attribution (evaluations attributed to others about one's body and appearance). The subscales have high internal consistency and 3 month test-retest reliability [35].

### Statistical analyses

Descriptive statistics (means ± SD, or median and interquartile ranges, as appropriate) will be used to describe the study sample with regard to baseline characteristics. To evaluate the effects of the intervention, longitudinal linear and logistic regression analysis will be used. The mean difference between intervention and control group with 95%-confidence intervals will be calculated. Regression analyses will be adjusted for baseline values and possible confounders such as gender and age. Also effect modification will be investigated using interaction terms between intervention group and gender and age, respectively. All analyses will be performed according to the intention-to-treat-principle, meaning that we will not impute values for missing data [36].

### Economic evaluation

An economic evaluation will be conducted from a societal perspective. All relevant costs will be assessed, valued and analysed. Costs will include costs of the interventions (Go4it programme and usual care), but also other health care related costs. For example, costs of visits to general practitioners, psychologists, physiotherapists, dieticians, paediatricians will also be estimated as well as costs of medication. Indirect costs of production loss are not relevant in this population of teenagers. Relevant cost data will be collected through cost diaries [37]. Costs will be valued according to guidelines for economic evaluation in the Netherlands. Primary outcomes in the economic evaluation will be quality of life, BMI, physical activity and dietary behaviour. The economic evaluation will be performed according to the intention-to-treat principle. A cost-effectiveness analysis will be performed. The mean differences in total costs between intervention groups will be estimated and bootstrapping will be used to estimate 95% confidence intervals.

Cost-effectiveness ratios will be estimated using bootstrapping techniques and graphically presented on cost-effectiveness planes. The incremental cost-effectiveness ratio expresses the additional costs of the Go4it programme per unit improvement of effect. Acceptability curves will also be presented in which the probability that the programme is cost-effective is expressed as a function of willingness-to-pay.

## Discussion

The Go4it program will be evaluated in a randomised controlled trial including an economic evaluation. We will determine the effectiveness and cost-effectiveness of this program, regarding body composition, energy requirement, dietary and physical activity behaviour, quality of life and body self esteem. The results of this study contribute to the evidence based knowledge of how to improve dietary, physical activity and sedentary behaviour among obese adolescents and how these behaviours affect weight and the development of Diabetes Mellitus type 2.

## Competing interests

The authors declare that they have no competing interests.

## Authors' contributions

AH, MC, PW, MT and HD provided support in the design of the study and contributed input into the main ideas of this paper. AH drafted the manuscript. MC, PW, MT and HD provided support during the development of the intervention. All authors contributed to the further writing of the manuscript. All authors read and approved the final manuscript.

## Pre-publication history

The pre-publication history for this paper can be accessed here:



## References

[B1] Dietz WH (1998). Health consequences of obesity in youth: childhood predictors of adult disease. Pediatrics.

[B2] Schwimmer JB, Burwinkle TM, Varni JW (2003). Health-related quality of life of severely obese children and adolescents. JAMA.

[B3] Sinha R, Fisch G, Teague B, Tamborlane WV, Banyas B, Allen K, Savoye M, Rieger V, Taksali S, Barbetta G, Sherwin RS, Caprio S (2002). Prevalence of impaired glucose tolerance among children and adolescents with marked obesity. N Engl J Med.

[B4] Singh AS, Mulder C, Twisk JWR, van Mechelen W, Chinapaw MJM (2008). Tracking of childhood overweight into adulthood: a systematic review of the literature. Obes Rev.

[B5] Hofsteenge GH, Weijs PJM, Wagenaar K, Chin A Paw MJM, Delemarre-van der Waal HA (2008). [First favourable results of the group education programme Go4it for overweight and obese adolescents][Article in Dutch]. Ned Tijdschr Geneeskd.

[B6] Doak CM, Visscher TLS, Renders CM, Seidell JC (2006). The prevention of overweight and obesity in children and adolescents: a review of interventions and programmes. Obes Rev.

[B7] Summerbell CD, Ashton V, Campbell KJ, Edmunds L, Kelly S, Waters E (2003). Interventions for treating obesity in children. Cochrane Database Syst Rev.

[B8] Summerbell CD, Waters E, Edmunds LD, Kelly S, Brown T, Campbell KJ (2005). Interventions for preventing obesity in children. Cochrane Database Syst Rev.

[B9] Jelalian E, Saelens BE (1999). Empirically supported treatments in pediatric psychology: pediatric obesity. J Pediatr Psychol.

[B10] Wilson P, O'Meara S, Summerbell C, Kelly S (2003). The prevention and treatment of childhood obesity. Qual Saf Health Care.

[B11] Braet C, Tanghe A, Decaluwe V, Moens E, Rosseel Y (2004). Inpatient treatment for children with obesity: weight loss, psychological well-being, and eating behavior. J Pediatr Psychol.

[B12] Renders CM, Delemarre-van de Waal HA, Dekker JM, Hirasing RA (2003). Insulineresistentie en diabetes mellitus type 2 bij kinderen met overgewicht. Ned Tijdschr Geneeskd.

[B13] Epstein LH, Valoski A, Wing RR, McCurley J (1994). Ten-year outcomes of behavioral family-based treatment for childhood obesity. Health Psychol.

[B14] Singh AS, Chin AP, Kremers SPJ, Visscher TLS, Brug J, van Mechelen W (2006). Design of the Dutch Obesity Intervention in Teenagers (NRG-DOiT): systematic development, implementation and evaluation of a school-based intervention aimed at the prevention of excessive weight gain in adolescents. BMC Public Health.

[B15] Cole TJ, Bellizzi MC, Flegal KM, Dietz WH (2000). Establishing a standard definition for child overweight and obesity worldwide: international survey. British Medical Journal.

[B16] Achenbach TM, EC (1983). Manual for the child behavior checklist and revised child behavior profile.

[B17] Verhulst FC, Koot JM, Akkerhuis GW, Veerman JW (1990). Praktische handleiding voor de CBCL.

[B18] Achenbach TM (1991). Manual for the Youth Self-Report and 1991 Profile.

[B19] Achenbach TM, EC (1983). Manual for the Youth Self-Report and profile.

[B20] Fairburn CG, Beglin SJ (1994). Assessment of eating disorders: interview or self-report questionnaire?. Int J Eat Disord.

[B21] Fairburn CG, Cooper Z, CG Fairburn, GT Wilson (1993). The Eating Disorder Examination. Binge eating: Nature, Assessment, and treatment.

[B22] Ellis KJ (2000). Human body composition: In vivo methods. Physiological Reviews.

[B23] Lewy VD, Danadian K, Arslanian S (1999). Determination of body composition in African-American children: Validation of bioelectrical impedence with dual energy X-ray absorptiometry. J Pediatr Endocrinol Metab.

[B24] Weir JBD (1949). New methods for calculating metabolic rate with special reference to protein metabolism. J Physiol.

[B25] American Diabetes Association (2006). Diagnosis and classification of diabetes mellitus. Diabetes Care.

[B26] van Assema P, Brug J, Ronda G, Steenhuis I (2001). The relative validity of a short Dutch questionnaire as a means to categorize adults and adolescents to total and saturated fat intake. J Hum Nutr Diet.

[B27] Van Assema P, Brug J, Ronda G, Steenhuis I, Oenema A (2002). A short dutch questionnaire to measure fruit and vegetable intake: relative validity among adults and adolescents. Nutr Health.

[B28] van Strien T, Frijters JER, Bergers GPA, Defares PB (1986). The Dutch eating Behavior Questionnaire (DEBQ) for assessment of restrained, emotional and external eating behavior. International Journal of Eating Disorders.

[B29] van Strien T (2002). Dutch Eating Behavior Questionnaire manual.

[B30] Wendel-Vos GCW, Schuit AJ, Saris WHM, Kromhout D (2003). Reproducibility and relative validity of the short questionnaire to assess health-enhancing physical activity. J Clin Epidemiol.

[B31] Landgraf JM, Abetz L, Ware JE (1996). The CHQ User's Manual.

[B32] Landgraf JM, Abetz LN (1996). Functional status and well-being of children representing three cultural groups: Initial self-reports using the CHQ-CF87.

[B33] Varni JW, Seid M, Kurtin PS (2001). PedsQL 4.0: reliability and validity of the Pediatric Quality of Life Inventory version 4.0 generic core scales in healthy and patient populations. Med Care.

[B34] Varni JW, Seid M, Knight TS, Uzark K, Szer IS (2002). The PedsQL 4.0 Generic Core Scales: sensitivity, responsiveness, and impact on clinical decision-making. J Behav Med.

[B35] Mendelson BK, Mendelson MJ, White DR (2001). Body-esteem scale for adolescents and adults. J Pers Assess.

[B36] Hollis S, Campbell F (1999). What is meant by intention to treat analysis? Survey of published randomised controlled trials. BMJ.

[B37] Goossens ME, Rutten-van Molken MP, Vlaeyen JW, Linden SM van der (2000). The cost diary: a method to measure direct and indirect costs in cost-effectiveness research. J Clin Epidemiol.

